# No major impact of prescribed CAD drugs on myocardial perfusion uptake derived by [^82^]rubidium PET

**DOI:** 10.1007/s12350-021-02786-5

**Published:** 2021-10-05

**Authors:** Rudolf A. Werner, Steven P. Rowe, Takahiro Higuchi

**Affiliations:** 1 Department of Nuclear Medicine, University Hospital Würzburg, Oberdürrbacherstr. 6, 97080 Würzburg, Germany; 2 The Russell H Morgan Department of Radiology and Radiological Sciences, Johns Hopkins School of Medicine, Baltimore, USA; 3 Okayama University Graduate School of Medicine, Dentistry and Pharmaceutical Sciences, Okayama, Japan

Precise assessment of myocardial perfusion and blood flow is critical in patients with coronary artery disease (CAD), paving the way to identify subjects that will most likely benefit from coronary intervention.^1^ Various imaging modalities have been proposed to provide guidance in regards to those patients that would benefit from cardiac catheterization.^2^,^3^ For instance, single photon emission computed tomography (SPECT) has been established for this purpose, and recent developments such as cardiocentric acquisitions, or cadmium-zinc-telluride detectors, have provided an even more reliable read-out of the status of myocardial perfusion.^3^,^4^

Furthermore, given its tremendous success in oncology with more and more devices installed,^5^–^7^ an increasing number of facilities are using positron emission tomography (PET) for cardiac imaging.^8^ In this regard, [^82^]Rubidium ([^82^]Rb) PET myocardial perfusion imaging (MPI) has been widely utilized for decades^9^ due to availability from on-site production using a cyclotron.^3^,^10^ Further advantages relative to conventional SPECT MPI, include, but are not limited to, low radiation exposure, measurement of myocardial blood flow (coronary flow reserve) or convenient rest-stress protocols within 30 minutes.^10^

Given the increased use of [^82^]Rb^10^ or other radiotracers used in nuclear cardiology,^11^ it is important to ensure that any potential impact of prescribed drugs on myocardial uptake is understood. For instance, cardiac nerve integrity assessments using [^123^I]-metaiodobenzylguanidine can be substantially altered by concomitant drug intake,^12^ increasing the risk of false-positive or false-negative findings when such scans are interpreted. Given the high rate of [^82^]Rb PET scans conducted in the clinic, Bentsen et al aimed to determine whether drugs used to treat CAD may affect the [^82^]Rb distribution in the myocardium.^13^ Such studies in humans would be desirable, but a well-designed trial cannot be rigorously executed,^12^ as individuals with CAD will most likely not tolerate withdrawal of their life-saving medication(s).^1^

Therefore, the authors of the present study conducted a thorough investigation in rats, which were treated by a sophisticated protocol, including various drugs also prescribed in the clinic (e.g., amiodarone, acetylsalicylic acid, or metoprolol succinate).^1^ All animals were imaged on day 1 and day 7 and rats were compared to treatment-naïve controls by analyzing standardized uptake values (SUV). Of note, no relevant impact on cardiac uptake by any of the investigated drugs was noted, except for amiodarone significantly reducing SUV ratio from baseline to follow-up.^13^ In a clinical setting, these findings are relevant, as the investigated drugs are also endorsed by current guidelines.^1^

In principle, the nuclear cardiologist or molecular imaging expert interpreting [^82^]Rb MPI scans can be certain that the concomitant intake of medication at the date of scan will most likely not alter the derived imaging results. However, one may speculate whether [^82^]Rb kinetic parameters assessing absolute myocardial blood flow in patients treated with amiodarone could be substantially affected. Therefore, despite its difficulties to perform a human trial, further investigations in patients under amiodarone are warranted. As an antiarrhythmic drug (AAD) used for life-threatening ventricular tachycardia, amiodarone has an inhibitory effect on the Na^+^/K^+^-ATPase pump,^14^ which provides a possible explanation for an inhibition of [^82^]Rb uptake in the heart as this radiotracer enters cardiomyocytes through exactly the same target mechanism.^15^ Amiodarone has been suggested as the most potent AAD to restore rhythm (SR) in atrial fibrillation (AF), but dofetilide is also used for this purpose.^16^ Not surprisingly, both drugs are prescribed for SR restoration in AF with an ejection fraction of ≤ 35%.^17^,^18^ As such, dofetilide should also be investigated in the setting described by Bentsen et al.

Nonetheless, the effect of amiodarone on [^82^]Rb uptake is of importance, as this drug is the most commonly prescribed medication for AF.^17^ Despite the numerous advantages of [^82^]Rb,^10^ [^18^F]flurpiridaz PET for MPI has been subject to research in recent years, as its longer half-life of 110 minutes may allow for more flexibility in imaging protocols, in particular in a busy nuclear cardiology practice.^19^–^21^ Therefore, such studies as the one presented by Bentsen et al should also be repeated by using [^18^F]flurpiridaz,^13^ preferably with a coronary occlusion model compared to treated vs. treatment-naïve controls.
